# Management of stage 1 renal cell cancer in patients immunosuppressed for organ transplantation or autoimmune disease

**DOI:** 10.3389/fruro.2023.1324696

**Published:** 2023-12-08

**Authors:** Ali Ghasemzadeh, Eric T. Wendt, Brendan Dolan, Juliana Craig, Glenn O. Allen, E. Jason Abel, Daniel D. Shapiro

**Affiliations:** ^1^ Department of Urology, University of Wisconsin School of Medicine and Public Health, Madison, WI, United States; ^2^ Division of Urology, William S. Middleton Memorial Veterans Hospital, Madison, WI, United States

**Keywords:** kidney cancer, renal cell carcinoma, immunosuppression, active surveillance, transplantation

## Abstract

**Objective:**

To describe the treatment and outcomes of patients who are medically immunosuppressed due to prior organ transplantation or autoimmune disease with clinical T1 renal cell carcinoma (cT1).

**Methods:**

An institutional database of patients treated for RCC was queried for patients with cT1 RCC and on chronic medical immunosuppression at the time of RCC diagnosis. The outcomes for patients undergoing (1) surgery, (2) ablation, or 3) active surveillance (AS) are described.

**Results:**

Between 2010 and 2022, 74 medically immunosuppressed patients with RCC were identified and treated using surgery (*n* = 29), ablation (*n* = 33), or AS (*n* = 12). Seven (58%) AS patients underwent deferred treatment (six ablations and one nephrectomy) due to tumor growth. For surgery patients and ablation patients, the 30-day readmission rates [17% and 9%, respectively (*p* = 0.7)], and 90-day complication rates [24% and 21%, respectively (*p* = 0.9)] were similar. One (3%) surgical patient and two (6%) ablation patients recurred locally. Despite being immunosuppressed, only one (3%) surgical patient, one (3%) ablation patient, and no AS patients progressed to metastatic disease. No significant differences were noted for the local recurrence-free rates, metastasis-free rates, and overall survival for the three cohorts (*p* > 0.05 for all).

**Conclusions:**

Patients with stage one RCC with medical immunosuppression can be safely managed through surgery, thermal ablation, or active surveillance, with similar outcomes to historical series of non-immunosuppressed patients. Future prospective studies should investigate shared decision making in this patient cohort and include discussion of less aggressive options that minimize morbidity but preserve oncologic control.

## 1 Introduction

Immunosuppression is necessary following organ transplantation and for the treatment of certain autoimmune diseases (1) and may result in significant morbidity (2, 3). Prior population-based studies have demonstrated that cancer occurrence is increased in patients who are chronically immunosuppressed (4), and a five-to-seven-fold increase in the incidence of kidney cancer has been observed in solid organ transplant patients specifically (4–6). The mechanisms underlying this are unknown but may be related to reduced immune surveillance with chronic immunosuppression. Additionally, patients with chronic kidney disease in their native kidneys frequently develop cystic degenerative changes and are predisposed to developing RCC, with an increased incidence of papillary RCC compared with the general population (7).

Patients presenting with cT1 (i.e., with tumors measuring <7 cm confined to the kidney) RCC who are medically immunosuppressed have multiple treatment options. Treatment decisions are evaluated in the context of a patient’s comorbidity, which may be substantial among immunosuppressed patients (8), in addition to their risk of progression to metastatic RCC, which is not well defined in immunosuppressed populations. Nephrectomy can be considered, with radical nephrectomy preferred in the native kidneys among patients with end-stage renal disease (ESRD) and partial nephrectomy preferred in transplant allografts. Ablation may also be considered, but ablation is known to have a slightly higher local recurrence rate among non-immunosuppressed patients (9). Concern regarding a greater rate of recurrence and progression among immunosuppressed patients than in the general population may influence treatment choice; however, little data are available to support this assumption. Active surveillance has been increasingly utilized for cT1a RCC in the modern era, which has excellent outcomes compared with surgery (10), but may not be offered to medically immunosuppressed patients because of a concern for more aggressive tumor behavior. Due to the relative scarcity of patients who are immunosuppressed and have cT1 RCC, there are few studies evaluating treatment outcomes. However, understanding these outcomes is critical to improving treatment options for stage 1 RCC patients treated with medical immunosuppression. The objective of this study was to examine the clinical and perioperative outcomes of medically immunosuppressed patients with cT1 RCC that were treated using surgery, ablation, or active surveillance.

## 2 Materials and methods

After institutional review board approval, a prospectively maintained RCC database was queried and clinical and pathological data from 2010–2022 were reviewed and collected for patients who were treated using surgery, thermal ablation, or active surveillance for RCC. Patients were included if they had cT1 RCC tumors and were on chronic immunosuppression, which was defined as the use of a selective immunosuppressant (as classified by the WHO) or prednisone at a dose of ≥ 5 mg for at least 3 months prior to their treatment for RCC and continuing throughout follow-up. **Table 1** summarizes the immunosuppressive medications that were considered in this study. It was our institutional protocol to continue all immunosuppressive medications through the pre- and postoperative periods without changing the regimen or dose. Patients were considered as part of the active surveillance cohort if they underwent surveillance for at least 6 months from renal mass diagnosis to intervention. Only patients with a pathologic diagnosis of RCC (obtained either through biopsy or surgical specimen) were included in this study. Patients with metastatic disease at the time of diagnosis were excluded.

**Table 1:** List of immunosuppressant medications considered in this study.

Medication	Class	Mechanism of action
Prednisone	Glucocorticoid	Modulation of gene expression across multiple targets leading to immunosuppression
Adalimumab	Monoclonal Antibody	TNF-α targeting antibody
Azathioprine	Antimetabolite	Purine synthesis inhibitor
Cyclosporine	Calcineurin Inhibitor	Inhibition of T-cell activation
Tacrolimus	Calcineurin Inhibitor	Inhibition of T-cell activation
Mesalazine	Aminosalicylate	Modulation of cyclooxygenase and lipoxygenase pathways
Sulfasalazine	Aminosalicylate	Modulation of cyclooxygenase and lipoxygenase pathways
Everolimus	mTOR inhibitor	Inhibition of T-cell activation
Rituximab	Monoclonal Antibody	CD20 targeting antibody leading to B-cell depletion
Methotrexate	Antimetabolite	Inhibition of thymidine synthesis
Mycophenolate Mofetil	Antimetabolite	Purine synthesis inhibitor

The data points pertaining to intervention outcomes were collected. Postoperative creatinine was defined as the creatinine value measured a minimum of 90 days post procedure. Charts were reviewed for follow-up dates, and the 30-day readmissions and 90-day complications were evaluated for the surgery and ablation cohorts. Complications were graded using the Clavien–Dindo complication grading system (11). Local recurrence was considered for partial nephrectomy patients if a tumor developed in the patients’ ipsilateral kidney after at least one negative cross-sectional imaging (either computed tomography [CT] or magnetic resonance imaging [MRI]). Tumors that underwent ablation were considered to have local recurrence if the cross-sectional images demonstrated no enhancement in the ablation zone during initial follow-up, and the subsequent images demonstrated either an enhancing mass in the ablation zone or a growing mass among patients who could not undergo a contrasted study. Our institutional ablation practice is to perform microwave ablation as previously described (12). Patients were followed up using cross-sectional imaging (either CT or MRI) of the chest, abdomen, and pelvis, and labs, regardless of the treatment approach, every 6 months for the first 3 years, and yearly thereafter. For patients who underwent radical nephrectomy, local recurrence was only considered for growing or enhancing soft tissue masses arising within the renal fossa. Metastatic progression was defined as histologically proven RCC masses in any lymph nodes, visceral structures or soft tissues (soft tissues outside the renal fossa in cases of radical nephrectomy).

Summary statistics were reported and compared among the three treatment cohorts using Wilcoxon rank sum or Fisher’s exact tests. The survival analysis, recurrence, and metastasis-free survival were evaluated and plotted on Kaplan–Meier curves. The survival outcomes were compared using the logrank test. The statistical significance was defined as a *p*-value < 0.05.

## 3 Results

### 3.1 Patient demographics and tumor characteristics

From 2010 to 2022, we identified 74 patients with cT1 RCC who were medically immunosuppressed. The reasons for immunosuppression are listed in **Table 2**. The most common indication for medical immunosuppressed was organ transplantation (61 out of 74 patients, 82%). The largest group of immunosuppressed patients was composed of patients who had previously undergone a kidney transplant. In this group, most patients received surgical treatment for their RCC, followed by ablation and active surveillance (62%, 39%, and 33%, respectively). Patients with RCC in a transplant allograft kidney, however, were more likely to undergo ablation or AS than surgery (40%, 57%, and 4%, respectively). Only a small number of patients had simultaneous multi-organ transplants (10 out of 74 patients, 13.5%). Among patients on immunosuppressive regimens due to rheumatologic disorders, none underwent surgery, opting for ablation (*n* = 6) or active surveillance (*n* = 2) instead.

**Table 2:** Conditions requiring chronic medical immunosuppression.

Immunosuppression indication	*n*	Surgery, *n* = 29	Ablation, *n* = 33	Active surveillance, *n* = 12
Kidney transplant	35 (47)	18 (62)	13 (39)	4 (33)
Liver transplant	6 (8)	2 (7)	3 (9)	1 (8)
Bone marrow transplant	4 (5)	1 (3)	2 (6)	1 (8)
Kidney and liver transplant	3 (4)	2 (7)	1 (3)	0
Kidney and pancreas transplant	6 (8)	3 (10)	0	3 (25)
Lung transplant	4 (5)	1 (3)	2 (6)	1 (8)
Heart transplant	2 (3)	2 (7)	0	0
Lung and kidney transplant	1 (1)	0	1 (3)	0
Rheumatologic disorder, chronic steroids	12 (16)	0	10 (30)	2 (17)
Inflammatory bowel disease, chronic steroids	1 (1)	0	1 (3)	0

In terms of treatment (**Table 3**), 29 out of 74 (39%) patients underwent surgery, 33 out of 74 (45%) underwent ablation, and 12 out of 74 (16%) initially pursued active surveillance (AS). The patients who received surgical treatment were younger than those who underwent ablation or active surveillance (*p* < 0.002, surgery vs ablation; *p* < 0.02, surgery vs AS). Most patients were male. There was no difference among the treatment groups based on patient race. The Eastern Cooperative Oncology Group (ECOG) performance status was similar among all groups; however, only the ablation and AS cohorts had patients with an ECOG performance status ≥ 2. The patients who underwent surgery had fewer comorbidities than patients who underwent ablation or AS based on Charlson comorbidity index scores.

**Table 3:** Clinical and pathological variables for medically immunosuppressed patients undergoing surgical, ablative, or active surveillance treatments.

Clinical and pathological variables	Surgery *n* = 29	Ablation *n* = 33	Active surveillance *n* = 12	*p-*value (surgery vs ablation)	*p-*value (surgery vs AS)	*p-*value (ablation vs AS)
Median age, years (IQR)	51 (41 to 63)	64 (56 to 68)	63 (56 to 69)	0.002	0.02	0.9
Gender, *n* (%)				0.8	0.3	0.5
Male	18 (62)	22 (67)	10 (83)			
Female	11 (38)	11 (33)	2 (17)			
Race, *n* (%)				0.4	1	0.5
White	22 (76)	29 (88)	9 (75)			
Black	5 (17)	2 (6)	2 (17)			
Other	2 (7)	2 (6)	1 (8)			
ECOG, *n* (%)				1	0.4	0.8
0	23 (79)	26 (79)	9 (75)			
1	6 (21)	6 (18)	2 (17)			
≥2	0	1 (3)	1 (8)			
CCI, *n* (%)				0.007	0.003	0.3
0–3	23 (79)	14 (42)	3 (25)			
4–6	4 (14)	15 (46)	5 (42)			
≥7	2 (7)	4 (12)	4 (33)			
Median BMI, kg/m^2^ (IQR)	28.4 (23.1 to 33.4)	29.8 (27.7 to 36.8)	26.5 (19.8 to 32.2)	0.054	0.3	0.02
Grade, *n* (%)				0.01	0.04	0.2
1–2	19 (65)	25 (76)	12 (100)			
3–4	8 (28)	1 (3)	0			
Not graded	2 (7)	7 (21)	0			
RCC subtype, *n* (%)				0.6	0.3	0.5
Clear cell	15 (52)	19 (58)	7 (58)			
Papillary	8 (28)	11 (33)	5 (42)			
Chromophobe	2 (7)	3 (9)	0			
RCC unclassified	3 (10)	0	0			
Clear cell papillary	1 (3)	0	0			
cTstage, *n* (%)				0.001	1	0.02
cT1a	21 (72)	33 (100)	9 (75)			
cT1b	8 (28)	0	3 (25)			
Median max radiographic tumor diameter, cm (IQR)	3.15 (1.85 to 5.1)	2.3 (1.9 to 2.9)	3.05 (2.35 to 4.7)	0.06	0.8	0.08
Median pathologic size, cm (IQR)	2 (1.6 to 4.3)	–	–			
RCC in transplanted kidney, *n* (%)*	1 (4)	6 (40)	4 (57)	0.01	0.006	0.7

IQR, Interquartile Range; ECOG, Eastern Cooperative Oncology Group Performance Status; CCI, Charlson Comorbidity Index; BMI, Body Mass Index; RCC, Renal Cell Carcinoma. *Rates of RCC in transplanted kidney are compared to those who had kidney transplants.

RCC histologic type was similar across treatment groups with clear cell RCC being most common. There was, however, an overrepresentation of papillary histology compared with population-based series (13). In general RCC cohorts, papillary RCC comprises 10%–15% of RCC diagnoses. In our immunosuppressed population, 24 out of 74 (32%) had papillary RCC. Histologic grade was examined, and a greater proportion of grade 3–4 tumors were seen in patients who underwent surgery (*p* = 0.01, surgery vs ablation; *p* = 0.04, surgery vs AS). Similar proportions of patients with cT1b masses underwent surgery and AS, 28% vs 25%, respectively (*p* = 1). All patients who underwent ablation had cT1a masses. No difference was seen in the size of the tumors among the groups.

### 3.2 Perioperative outcomes of immunosuppressed patients with RCC undergoing surgery and ablation

Among patients who underwent upfront surgery, 86% had radical nephrectomy and 14% had partial nephrectomy (**Table 4**). Among the upfront ablation cohort, the majority underwent microwave ablation (28 out of 33 patients, 85%), with the remaining patients undergoing cryoablation (five out of 33, 15%) Intraoperative complications were rare, with none occurring in patients undergoing surgery and only one among patients undergoing ablation. In patients who had surgery or ablation, there was no difference in the rates of postoperative transfusion or embolization. The 30-day readmission and 90-day complication rates were similar between surgery and ablation patients (**Table 5**). In our cohort of immunosuppressed patients, we did not see any wound complications in patients treated using surgery or ablation. There were no deaths within 90 days of the procedure date for any group. The median change in creatinine level from the preoperative to the postoperative periods was similar for the surgery vs ablation cohorts, suggesting that the higher postoperative creatinine levels of surgery patients reflect higher preoperative creatinine levels, though this difference was not statistically significant.

**Table 4:** Perioperative outcomes of immunosuppressed patients with renal cell carcinoma undergoing surgery and ablation.

Intervention outcomes	Surgery *n* = 29	Ablation *n* = 33	*p*-value
Median estimated blood loss, mL (IQR)	100 (50–150)	–	
Nephrectomy type, *n* (%)			
Radical	25 (86)	–	
Partial	4 (14)	–	
Ablation type, *n* (%)			
Microwave	–	28 (85)	
Cryoablation	–	5 (15)	
Intraoperative complication, *n* (%)	0	1 (3)	0.056
Postoperative transfusion, *n* (%)	4 (14)	3 (9)	0.7
Postoperative embolization, *n* (%)	0	2 (6)	0.5
Length of hospitalization, days	3 (2–5)	1 (1–1)	<0.001
Median preoperative creatinine level, mg/dL (IQR)	1.5 (0.99 to 2.06)	1.2 (0.93 to 1.67)	0.08
Median postoperative creatinine level, mg/dL (IQR)	1.73 (1.3 to 2.2)	1.17 (0.86 to 1.71)	0.006
Median change in creatinine level, mg/dL (IQR)	0.1 (−0.97 to 0.36)	0.1 (−0.11 to 0.18)	0.2

IQR, interquartile range.

**Table 5:** Readmissions and complications following surgery or ablation in immunosuppressed patients.

Intervention outcomes	Surgery *n* = 29	Ablation *n* = 33	*p*-value
30-day readmission, *n* (%)	5 (17)	3 (9)	0.7
Fluid overload	1 (3)	0	
Myocardial Infarction	1 (3.5)	0	
Bowel injury	2 (7)	0	
Upper respiratory infection	1 (3.5)	0	
Urinary tract infection	0	1 (3)	
Acute kidney injury	0	1 (3)	
Renal hemorrhage	0	1 (3)	
90-day complication, *n* (%)	4 (14)	8 (24)	0.4
Infectious complication	2 (7)	4 (12)	
Bleeding leading to transfusion	1 (3)	3 (9)	
Transplant allograft rejection	1 (3)	0	
Acute kidney injury	0	1 (3)	
Clavien grade, *n* (%)			0.2
I–II	3 (10)	8 (24)	
III–V	1 (3)	0	
90-day mortality, *n* (%)	0	0	1

A subset of patients undergoing active surveillance went on to be treated using surgery or ablation (*n* = seven out of 12). One patient underwent radical nephrectomy, and six patients underwent microwave ablation. In patients on AS that went on to deferred treatment, the average time on AS was 1.3 years. The perioperative complication rates in this patient group were similar to those who were treated with surgery or ablation upfront (**Table 6**).

**Table 6:** Perioperative outcomes of immunosuppressed patients with renal cell carcinoma who initially underwent active surveillance followed by deferred treatment.

Intervention outcomes	Deferred surgery *n* = 1	Deferred ablation *n* = 6
Median estimated blood loss, mL (IQR)	150	–
Nephrectomy type, *n* (%)		
Radical	1	–
Postoperative transfusion, *n* (%)	0	0
Postoperative embolization, *n* (%)		
Intraoperative complication, *n* (%)	0	1 (17)
Length of hospitalization, days	4	1 (1–2)
30-day readmission, *n* (%)	0	1 (17)
90-day complication, *n* (%)	1	1 (17)
Infectious complication	1	1 (17)
Clavien grade, *n* (%)		
I–II	1	0
III–V	0	1 (17)
90-day mortality, *n* (%)	0	0
Median preoperative creatinine level, mg/dL (IQR)	0.6	1.19 (1.05 to 1.75)
Median postoperative creatinine level, mg/dL (IQR)	0.78	1.46 (1.19 to 1.57)
Median change in creatinine level, mg/dL (IQR)	0.18	−0.08 (−0.11 to 0.36)

### 3.3 Clinical outcomes following surgery, ablation, and active surveillance

Over a follow-up period of more than 3 years, there were few local recurrences or progression to metastatic disease in patients treated with surgery, ablation, or active surveillance in our cohort (**Table 7**). More patients on active surveillance died during the follow-up period (*n* = 6, 50%) than did those who underwent surgery or ablation.

**Table 7:** Clinical outcomes by treatment cohort.

Survival analysis	Surgery *n* = 29	Ablation *n* = 33	Active surveillance *n* = 12
Median follow-up, months (IQR)	54 (37–74)	41 (21–79)	47 (22–85)
Local recurrence, *n* (%)	1 (3)	2 (6)	–
Progression to metastatic disease, *n* (%)	1 (3)	1 (3)	0
Site of metastasis	Paraaortic lymph nodes	Lung, bone	
Deceased, *n* (%)	3 (10)	9 (27)	6 (50)

Within the surgery cohort, one patient recurred in the nephrectomy bed and developed paraaortic nodal metastasis at 3 months following surgery and ultimately died of metastatic RCC 11 months after the index operation. In the ablation cohort, two patients developed local recurrence, both at the ablation zone. The first patient developed a recurrence 16 months after the initial ablation and was treated with radical nephrectomy. This patient subsequently developed metastatic disease of the lung and bone, but was treated with cabozantinib and remains in remission. The second patient developed recurrence 5 months after the index ablation and was treated using a second ablation, and ultimately died of leukemia 2 years later without any sign of RCC recurrence.

### 3.4 Survival following surgery, ablation, and active surveillance in medically immunosuppressed patients

**Figure 1:**
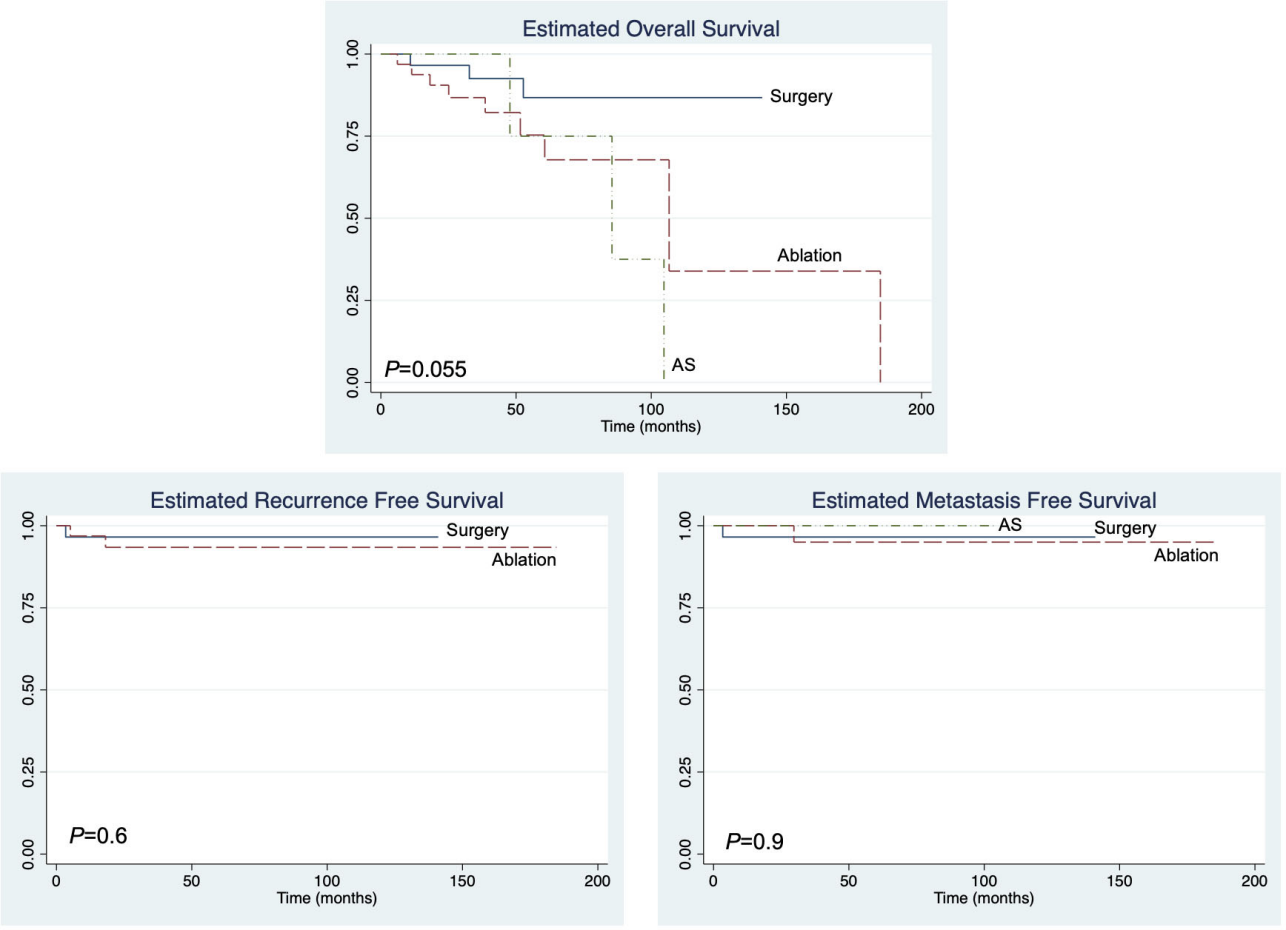
The overall recurrence-free and metastasis-free survival of immunosuppressed patients with T1 renal cell carcinoma.

We evaluated the overall (OS), recurrence-free (RFS), and metastasis-free (MFS) survival in our patient cohort. There was no statistically significant difference in the OS among the treatment groups (**Figure 1**). Additionally, no significant difference in RFS and MFS was seen among the treatment groups. Both recurrence and metastasis were rare events in our cohort, with only three out of 74 (4%) patients experiencing local recurrence and two out of 74 (3%) patients progressing to metastatic disease across the entire cohort.

## 4 Discussion

The optimal treatment of small renal masses in immunosuppressed patients is unknown and there is a theoretical risk of more aggressive disease due to reduced immune surveillance (14). In this study, we demonstrated that carefully selected immunosuppressed patients with pathologically confirmed cT1 RCC had similar outcomes to historical series of non-immunosuppressed patients with stage 1 RCC. Overall, disease recurrence and the metastatic progression of T1 RCC are relatively uncommon. In a recent large Swedish national registry study of 4,965 patients with non-metastatic cT1 RCC, over a follow-up period of more than 5 years, 11.6% of patients had recurrent disease. Most of the recurrences were progression to metastatic disease, with the most common sites being the bone and lung. Local disease recurrence was uncommon, occurring in only 1% of patients (15). The recurrence rates for patients treated using ablation were higher than for those treated using surgery. In a similar study of the same patient cohort with a focus on cT1a RCC, the disease recurrence rate after treatment was found to be 7.5% (16). In a large cohort of 1,539 patients with surgically treated small renal masses with a median follow-up period of 3 years, 2.4% of patients developed metastases (17). In our cohort of immunosuppressed patients with cT1 RCC over median follow-up periods of 4.5, 3.4, and 3.9 years for patients who had surgery, ablation, and active surveillance, respectively, only three out of 74 (4%) patients developed local recurrence after treatment, including two out of 74 (3%) patients who developed metastatic disease. These recurrence rates are similar to studies of non-immunosuppressed patients and suggest RCC tumor behavior may be similar in medically immunosuppressed patients. Importantly, this evidence argues against the notion that tumor behavior is significantly more aggressive in medically immunosuppressed patients.

While this study was not specifically designed to compare the survival among the treatment cohorts, we found no difference in OS, RFS, or MFS regardless of the treatment type over intermediate term follow-up periods of 54 months, 41 months, and 47 months in patients who underwent surgery, ablation, and AS, respectively. Among patients on AS, seven of 12 patients (58%) went on to deferred surgery (one patient, 14%) or ablation (six patients, 86%), with an average time to deferred treatment of 1.3 years. The published rates of delayed intervention in non-immunosuppressed patients with small renal masses indicate that the delayed intervention rate is between 10% and 30% (18–21). In our cohort, the delayed intervention rate was higher, but this likely reflects a selection bias for conversion from surveillance to treatment due to concern for potential tumor progression occurring while on active surveillance. Regardless of whether a patient is immunosuppressed or not, the careful follow-up of patients on AS is necessary to prevent disease progression. We demonstrated that, with appropriate follow-up active, active surveillance was safe in an immunosuppressed population, as indicated by a lack of progression to metastatic disease, and it should therefore still be considered an option for significantly comorbid patients with cT1 disease. To our knowledge, our data represent one of the first cohorts reporting active surveillance for cT1 RCC in the transplant and immunosuppressed patient population; thus, we are unable to compare our experience to that at other centers.

A large proportion of patients in our study were immunosuppressed after receiving a renal transplant for ESRD, and among renal transplant recipients, the majority of RCC tumors were identified within the native kidney. Only 24% of patients were identified with a RCC tumor within the transplant kidney. This aligns with published data demonstrating that the majority of RCC tumors in kidney transplant patients are found in native kidneys (22, 23). Interestingly, patients with ESRD are at higher risk for development of RCC and those on dialysis have a reported three-fold increased risk of RCC compared with non-dialysis-dependent patients (24). The etiology of this increased risk is not well understood, though there are several proposed mechanisms, including uremia-induced chronic inflammation, immunosuppression, oxidative stress, and shared disease processes driving both RCC and ESRD (25). It is unclear if immunosuppression itself or the associated underlying ESRD is the main causative factor leading to the initial development of RCC. In a study of over 200,000 kidney transplant candidates and recipients, the incidences of Kaposi sarcoma and lymphomas were increased during periods of improved renal function corresponding to post-transplant status, while the incidence of RCC increased during periods of reduced renal function, suggesting RCC development in this population may be driven by the physiologic changes associated with ESRD rather than the reduced immune surveillance associated with immunosuppression (26).

Similar to previous work, we found that a greater proportion of immunosuppressed patients had papillary histology (23, 27). Papillary histology appears to be enriched in this patient population, with different studies identifying between 20% and 60% of tumors as papillary (22, 23, 28–30). Similarly, 32% (24 out of 74) patients in our study had papillary histology RCC tumors. Most published series estimate the prevalence of papillary RCC in the non-immunosuppressed patient population to be 5%–10% (13). The mechanisms driving the increased prevalence of papillary RCC in this patient population are unknown but may be related to shared mechanisms driving ESRD, chronic inflammation, and oxidative stress (24). Additionally, acquired cystic kidney disease (ACKD) is a manifestation of ESRD and may result in papillary RCC with tumors being small, multifocal and possessing a papillary structure (31).

Both surgery and ablation appear safe, with few peri-procedural complications, despite a significantly comorbid population. We found no significant difference in the number of intraoperative complications or postoperative transfusion rates between the surgical and ablative treatment groups. We did not find that there was a significant difference in pre- and post-treatment renal function in patients treated using surgery or ablation. Similarly, in a multicenter study of transplant centers in France, the treatment of small renal masses using ablation or partial nephrectomy did not lead to a significant change in renal function, and no patient returned to dialysis following treatment (32). In a systematic review of thermal ablation for small renal masses in renal allografts, ablation was found to have low complication rates (< 10%) and induce no change in renal function (33). Most renal allograft ablation cases reported in the literature have used radiofrequency ablation. Our series represents the largest published renal allograft ablation experience with microwave ablation, showing it to be safe and comparable to radiofrequency ablation. Experience of the ablation team is important when endeavoring to perform renal allograft ablations. It is critical to understand the differences in renal perfusion, as transplant kidneys frequently have a reduced blood supply and smaller heat sink effect than normal kidneys. We advise that shorter-duration ablations are performed and that precautions to avoid collecting system injuries are taken, as these are less likely to heal or may result in strictures given the reduced blood supply and need for chronic immunosuppression. For patients with more centrally located tumors or tumors near the ureter, we cryoablation could also be considered.

Some limitations should be considered in the interpretation of our results. This was a retrospective study using data from experience at a high-volume renal cell carcinoma and organ transplant center. Thus, our patient population and results may not reflect those seen at other centers. While this is the largest series reported currently reported, only 74 patients were identified over a 12-year period, reflecting the rarity of patients with cT1 RCC and concomitant immunosuppression. Due to the retrospective nature of this study, we are unable to control differences in imaging surveillance schedules and follow-up protocols over time, which may have influenced the outcomes. Additionally, this study was designed as a purely observational study of patient outcomes and did not include a control cohort of non-immunosuppressed patients; thus, the study population was compared with previously reported series, which may contain populations with inherently different baseline characteristics.

## 5 Conclusion

Patients with stage one RCC who are medically immunosuppressed can be safely managed using surgery, ablation, or active surveillance. While the optimal management of T1 RCC in immunosuppressed patients remains unknown, our results demonstrate that all conventional treatment modalities appear safe with satisfactory oncologic outcomes, and may serve as a starting point for the development of specific clinical guidelines for the management of RCC among immunosuppressed patients.

## Data availability statement

The raw data supporting the conclusions of this article will be made available by the authors, without undue reservation.

## Ethics statement

The studies involving humans were approved by University of Wisconsin School of Medicine and Public Health. The studies were conducted in accordance with the local legislation and institutional requirements. Written informed consent for participation was not required from the participants or the participants’ legal guardians/next of kin in accordance with the national legislation and institutional requirements.

## Author contributions

AG: conceptualization, data curation, & writing – original draft, writing – review & editing. EW: conceptualization, data curation, formal analysis, & writing – original draft, writing – review & editing. BD: data curation, writing – review & editing. JC: writing – review & editing. GA: formal analysis & writing – review & editing. EA: conceptualization, methodology, & writing – original draft, writing – review & editing. DS: conceptualization, data curation, formal analysis, methodology, & writing – original draft, writing – review & editing.

## References

[1] Pilch NA Bowman LJ Taber DJ. Immunosuppression trends in solid organ transplantation: The future of individualization, monitoring, and management. Pharmacother: J Hum Pharmacol Drug Ther (2021) 41:119–31. doi: 10.1002/phar.2481 PMC8778961 33131123

[2] Cohen-Bucay A Gordon CE Francis JM. Non-immunological complications following kidney transplantation. F1000Research (2019) 8. doi: 10.12688/f1000research.16627.1 PMC6381799 30828430

[3] Sen A Callisen H Libricz S Patel B. Complications of solid organ transplantation cardiovascular, neurologic, renal, and gastrointestinal. Crit Care Clin (2019) 35:169–86. doi: 10.1016/j.ccc.2018.08.011 30447778

[4] Engels EA Pfeiffer RM Fraumeni JF Kasiske BL Israni AK Snyder JJ. Spectrum of cancer risk among US solid organ transplant recipients. Jama (2011) 306:1891–901. doi: 10.1001/jama.2011.1592 PMC3310893 22045767

[5] Collett D Mumford L Banner NR Neuberger J Watson C. Comparison of the incidence of Malignancy in recipients of different types of organ: A UK registry audit. Am J Transplant (2010) 10:1889–96. doi: 10.1111/j.1600-6143.2010.03181.x 20659094

[6] Karami S Yanik EL Moore LE Pfeiffer RM Copeland G Gonsalves L. Risk of renal cell carcinoma among kidney transplant recipients in the United States. Am J Transplant (2016) 16:3479–89. doi: 10.1111/ajt.13862 PMC5104677 27160653

[7] Dahle DO Skauby M Langberg CW Brabrand K Wessel N Midtvedt K. Renal cell carcinoma and kidney transplantation: A narrative review. Transplantation (2022) 106:e52–63. doi: 10.1097/TP.0000000000003762 PMC8667800 33741842

[8] Bottomley MJ Harden PN. Update on the long-term complications of renal transplantation. Br Med Bull (2013) 106:117–34. doi: 10.1093/bmb/ldt012 23645842

[9] Pierorazio PM Johnson MH Patel HD Sozio SM Sharma R Iyoha E. Management of renal masses and localized renal cancer: systematic review and meta-Analysis. J Urol (2016) 196:989–99. doi: 10.1016/j.juro.2016.04.081 PMC5593254 27157369

[10] Campbell SC Clark PE Chang SS Karam JA Souter L Uzzo RG. Renal mass and localized renal cancer: evaluation, management, and follow-up: AUA guideline: part I. J Urol (2021) 206:199–208. doi: 10.1097/JU.0000000000001911 34115547

[11] Dindo D Demartines N Clavien P-A. Classification of surgical complications. Ann Surg (2004) 240:205–13. doi: 10.1097/01.sla.0000133083.54934.ae PMC1360123 15273542

[12] Klapperich ME Abel EJ Ziemlewicz TJ Best S Lubner MG Nakada SY. Effect of tumor complexity and technique on efficacy and complications after percutaneous microwave ablation of stage T1a renal cell carcinoma: A single-Center, retrospective study. Radiology (2017) 284:160592. doi: 10.1148/radiol.2016160592 PMC5495130 28076721

[13] Bukavina L Bensalah K Bray F Carlo M Challacombe B Karam JA. Epidemiology of renal cell carcinoma: 2022 update. Eur Urol (2022) 82:529–42. doi: 10.1016/j.eururo.2022.08.019 36100483

[14] Griffith JJ Amin KA Waingankar N Lerner SM Delaney V Ames SA. Solid renal masses in transplanted allograft kidneys: A closer look at the epidemiology and management. Am J Transplant (2017) 17:2775–81. doi: 10.1111/ajt.14366 28544435

[15] Almdalal T Rosenblad AK Hellström M Kjellman A Lindblad P Lundstam S. Predictive characteristics for disease recurrence and overall survival in non-metastatic clinical T1 renal cell carcinoma – results from the National Swedish Kidney Cancer Register. Scand J Urol (2023) 57:67–74. doi: 10.1080/21681805.2022.2154383 36520023

[16] Almadalal T Sundqvist P Harmenberg U Hellström M Lindskog M Lindblad P. Clinical T1a renal cell carcinoma, not always a harmless disease—A national register study. Eur Urol Open Sci (2022) 39:22–8. doi: 10.1016/j.euros.2022.03.005 PMC9068725 35528783

[17] Kapur P Zhong H Araj E Christie A Cai Q Kim D. Predicting oncologic outcomes in small renal tumors. Eur Urol Oncol (2022) 5:687–94. doi: 10.1016/j.euo.2022.08.003 PMC9812257 36115820

[18] Gupta M Alam R Patel HD Semerjian A Gorin MA Johnson MH. Use of delayed intervention for small renal masses initially managed with active surveillance. Urol Oncol Semin Orig Investigations (2019) 37:18–25. doi: 10.1016/j.urolonc.2018.10.001 30446459

[19] Gupta M Jr MB Su L-M Crispen P. Delayed intervention of small renal masses on active surveillance. J Kidney Cancer VHL (2017) 4:24–30. doi: 10.15586/jkcvhl.2017.75 28725541 PMC5515897

[20] Crispen PL Viterbo R Boorjian SA Greenberg RE Chen DYT Uzzo RG. Natural history, growth kinetics, and outcomes of untreated clinically localized renal tumors under active surveillance. Cancer (2009) 115:2844–52. doi: 10.1002/cncr.24338 PMC2860784 19402168

[21] Abouassaly R Lane BR Novick AC. Active surveillance of renal masses in elderly patients. J Urol (2008) 180:505–9. doi: 10.1016/j.juro.2008.04.033 18550113

[22] Leveridge M Musquera M Evans A Cardella C Pei Y Jewett M. Renal cell carcinoma in the native and allograft kidneys of renal transplant recipients. J Urol (2011) 186:219–23. doi: 10.1016/j.juro.2011.03.032 21575970

[23] Tsivian M Caso JR Kimura M Polascik TJ. Renal tumors in solid organ recipients: Clinical and pathologic features. Urol Oncol Semin Orig Investigations (2013) 31:255–8. doi: 10.1016/j.urolonc.2010.11.006 21719326

[24] Hickman LA Sawinski D Guzzo T Locke JE. Urologic Malignancies in kidney transplantation. Am J Transplant (2018) 18:13–22. doi: 10.1111/ajt.14533 28985026

[25] Hu SL Chang A Perazella MA Okusa MD Jaimes EA Weiss RH. The nephrologist’s tumor: basic biology and management of renal cell carcinoma. J Am Soc Nephrol (2016) 27:2227–37. doi: 10.1681/ASN.2015121335 PMC4978061 26961346

[26] Yanik EL Clarke CA Snyder JJ Pfeiffer RM Engels EA. Variation in Cancer Incidence among Patients with ESRD during Kidney Function and Nonfunction Intervals. J Am Soc Nephrol (2016) 27:1495–504. doi: 10.1681/ASN.2015040373 PMC4849829 26563384

[27] Tsuzuki T Iwata H Murase Y Takahara T Ohashi A. Renal tumors in end-stage renal disease: A comprehensive review. Int J Urol (2018) 25:780–6. doi: 10.1111/iju.13759 30066367

[28] Filocamo MT Zanazzi M Marzi VL Guidoni L Villari D Dattolo E. Renal cell carcinoma of native kidney after renal transplantation: clinical relevance of early detection. Transplant Proc (2009) 41:4197–201. doi: 10.1016/j.transproceed.2009.08.082 20005368

[29] Moudouni SM Lakmichi A Tligui M Rafii A Tchala K Haab F. Renal cell carcinoma of native kidney in renal transplant recipients. BJU Int (2006) 98:298–302. doi: 10.1111/j.1464-410X.2006.06267.x 16879668

[30] Szmidt J Durlik M Gałązka Z Nazarewski S Górnicka B Ziarkiewicz-Wróblewska B. Low-stage renal carcinoma of the native kidneys in renal transplant recipients. Transplant Proc (2002) 34:583–4. doi: 10.1016/S0041-1345(01)02852-4 12009631

[31] Meola M Samoni S Petrucci I. Clinical scenarios in chronic kidney disease: kidneys’ Structural changes in end-stage renal disease. Contrib Nephrol (2016) 188:131–43. doi: 10.1159/000445475 27169876

[32] Tillou X Doerfler A Collon S Kleinclauss F Patard J ‐J. Badet L. *De novo* kidney graft tumors: results from a multicentric retrospective national study. Am J Transplant (2012) 12:3308–15. doi: 10.1111/j.1600-6143.2012.04248.x 22959020

[33] Favi E Raison N Ambrogi F Delbue S Clementi MC Lamperti L. Systematic review of ablative therapy for the treatment of renal allograft neoplasms. World J Clin cases (2019) 7:2487–504. doi: 10.12998/wjcc.v7.i17.2487 PMC6745334 31559284

